# Malnutrition as an independent risk factor for fluoropyrimidine- and oxaliplatin-based chemotherapy-induced cardiovascular toxicity in colorectal cancer: a single-center retrospective cohort study

**DOI:** 10.3389/fcvm.2026.1936804

**Published:** 2026-09-21

**Authors:** Xuyun Zhou, Biao Tang, Xiaomei Sheng, Haixia Chang

**Affiliations:** 1 Department of General Surgery, Shanghai Songjiang Sijing Hospital, Shanghai, China; 2 Department of General Surgery, Ezhou Central Hospital, Ezhou, Hubei, China; 3 Department of Warehouse, Jinling Hospital, Medical School of Nanjing University, Nanjing, Jiangsu, China; 4 Department of Nursing, The Fifth Affiliated Hospital of Xinjiang Medical University, Urumqi, Xinjiang, China

**Keywords:** cardio-oncology, cardiovascular toxicity, chemotherapy, colorectal cancer, fluoropyrimidine, malnutrition, NRS-2002, nutritional support

## Abstract

**Background:**

Fluoropyrimidine- and oxaliplatin-based regimens (FOLFOX/XELOX) are the standard of care for colorectal cancer (CRC) but carry significant cardiovascular toxicity. Malnutrition, present in up to 60% of post-surgical CRC patients, may amplify this toxicity through systemic inflammation and pharmacokinetic alterations; however, this relationship has not been specifically investigated.

**Methods:**

We conducted a retrospective cohort study of 320 CRC patients receiving FOLFOX or XELOX at the Shanghai songjiang sijing hospital (January 2020—December 2023). Nutritional status was assessed by the Nutritional Risk Screening 2002 (NRS-2002) and serum albumin. The primary endpoint was chemotherapy-induced cardiovascular toxicity events (CICVT), defined per 2022 ESC Cardio-Oncology Guidelines. Multivariable logistic regression, 1:1 propensity score matching (PSM), and receiver operating characteristic (ROC) analysis were performed. An intestinal fistula subgroup compared outcomes between total parenteral nutrition (TPN) and early enteral nutrition (EEN).

**Results:**

Nutritional risk (NRS-2002 ≥ 3) was identified in 112 patients (35.0%). CICVT occurred in 31.3% of patients at nutritional risk vs. 11.5% of well-nourished patients (*p* < 0.001). After PSM (89 matched pairs), malnutrition remained an independent risk factor (OR 2.93, 95% CI 1.41–6.07; *p* < 0.001). Multivariable analysis confirmed NRS-2002 ≥ 3 (adjusted OR 3.18, 95% CI 1.69–5.98; *p* < 0.001) and serum albumin < 35 g/L (adjusted OR 2.43, 95% CI 1.34–4.40; *p* = 0.003) as independent predictors. ROC AUC improved from 0.718 (NRS-2002 alone) to 0.764 when combined with albumin. Among 49 fistula patients, the TPN group had significantly higher CICVT rates than the EEN group (38.7% vs. 16.7%; *p* = 0.043).

**Conclusion:**

Malnutrition is an independent and modifiable risk factor for chemotherapy-induced cardiovascular toxicity in CRC. Integrating nutritional assessment into pre-chemotherapy cardiovascular risk stratification and prioritizing early enteral nutrition in fistula patients may mitigate cardiovascular morbidity.

## 1 Introduction

Colorectal cancer (CRC) is the third most commonly diagnosed cancer and the second leading cause of cancer-related mortality worldwide, with an estimated 1.93 million new cases and 935,000 deaths reported in 2020 (1). For patients with resectable or metastatic CRC, combination chemotherapy incorporating fluoropyrimidines [5-fluorouracil (5-FU) or capecitabine] with oxaliplatin — principally FOLFOX and XELOX regimens — constitutes the standard of care (2). Despite their demonstrated efficacy, these regimens carry a well-recognized burden of cardiovascular toxicity that can necessitate dose modification, treatment interruption, or discontinuation, thereby compromising oncological outcomes.

Fluoropyrimidine-induced cardiotoxicity (FIC) affects 1.2%–18% of treated patients depending on monitoring intensity and endpoint definitions (3, 4). The 2022 European Society of Cardiology (ESC) Guidelines on Cardio-Oncology classify 5-FU and capecitabine as moderate-risk cardiotoxic agents, mandating systematic pre-treatment cardiovascular risk assessment (5). Manifestations include coronary vasospasm, QTc prolongation, supraventricular arrhythmias, and — in severe cases — acute left ventricular dysfunction. Oxaliplatin additionally prolongs the QTc interval through voltage-gated sodium channel dysfunction and predisposes to hypomagnesemia-related arrhythmias (6). Bevacizumab, widely used in the metastatic setting, further augments cardiovascular risk, conferring a fourfold excess odds of Grade ≥ 3 hypertension and twofold excess odds of arterial thromboembolism compared with chemotherapy alone (7).

An underappreciated but clinically important determinant of cardiovascular toxicity in this population is nutritional status. Malnutrition affects 30%–60% of CRC patients at diagnosis and escalates substantially after surgical resection (8). Patients complicated by anastomotic leak or enterocutaneous fistula — affecting 3%–6% of CRC resections — face compounded nutritional adversity due to protein losses, systemic inflammation, and prolonged gastrointestinal dysfunction, frequently requiring parenteral nutritional support (9, 10), often guided by chronic intestinal failure management principles. Nutritional derangements, particularly hypoalbuminemia and elevated neutrophil-to-lymphocyte ratio (NLR), an established marker of systemic inflammation and oncological prognosis (11), may potentiate chemotherapy-related cardiovascular toxicity through three converging mechanisms: (i) altered drug pharmacokinetics with increased free-fraction exposure at the myocardial level under hypoproteinemic conditions (12); (ii) systemic inflammation-driven pro-oxidative and pro-apoptotic myocardial priming (13); and (iii) electrolyte deficiencies (hypomagnesemia, hypokalaemia) lowering the arrhythmia threshold and synergizing with oxaliplatin's repolarization-prolonging effects, consistent with drug-specific mechanistic data across platinum and fluoropyrimidine agents (14).

Despite the biological plausibility of these interactions, no study has specifically examined nutritional status as an independent risk factor for fluoropyrimidine-induced cardiovascular toxicity in CRC patients, and the cardiovascular implications of nutritional support strategies (enteral versus parenteral) in fistula patients proceeding to chemotherapy remain entirely unexplored. Addressing this gap is clinically urgent: unlike fixed patient characteristics, nutritional status is a modifiable target for pre-chemotherapy optimization.

This study therefore aimed to: (i) determine whether malnutrition (NRS-2002 ≥ 3; albumin < 35 g/L) is an independent predictor of CICVT in FOLFOX/XELOX-treated CRC patients; (ii) quantify the impact of nutritional support route (TPN vs. EEN) on cardiovascular outcomes in the post-surgical fistula subgroup; and (iii) evaluate the discriminative performance of nutritional parameters in combination for pre-chemotherapy cardiovascular risk stratification.

## 2 Methods

### 2.1 Study design and patient selection

This was a single-center retrospective cohort study conducted at the Department of General Surgery, Shanghai songjiang sijing hospital (January 2020–December 2023). Inclusion criteria: (1) age ≥ 18 years; (2) histopathologically confirmed CRC; (3) receipt of ≥ 2 cycles of FOLFOX (5-FU 400 mg/m2 IV bolus + 2400 mg/m2 46-hour infusion, leucovorin 400 mg/m2, oxaliplatin 85 mg/m2, every 2 weeks) or XELOX (capecitabine 1000 mg/m2 twice daily on days 1–14, oxaliplatin 130 mg/m2 on day 1, every 3 weeks); (4) available ECG and cardiac biomarker records at baseline and at each cycle. Exclusion criteria: (1) pre-existing cardiomyopathy or LVEF < 50% prior to chemotherapy; (2) prior anthracycline-based chemotherapy or mediastinal radiotherapy; (3) incomplete cardiac monitoring data; (4) follow-up < 3 months. The study was approved by the Institutional Review Board of the Shanghai songjiang sijing hospital (Approval No. 2023KYLWLL-ZXY). Patient consent was waived per institutional policy for retrospective studies (Figure 1).

**Figure 1:**
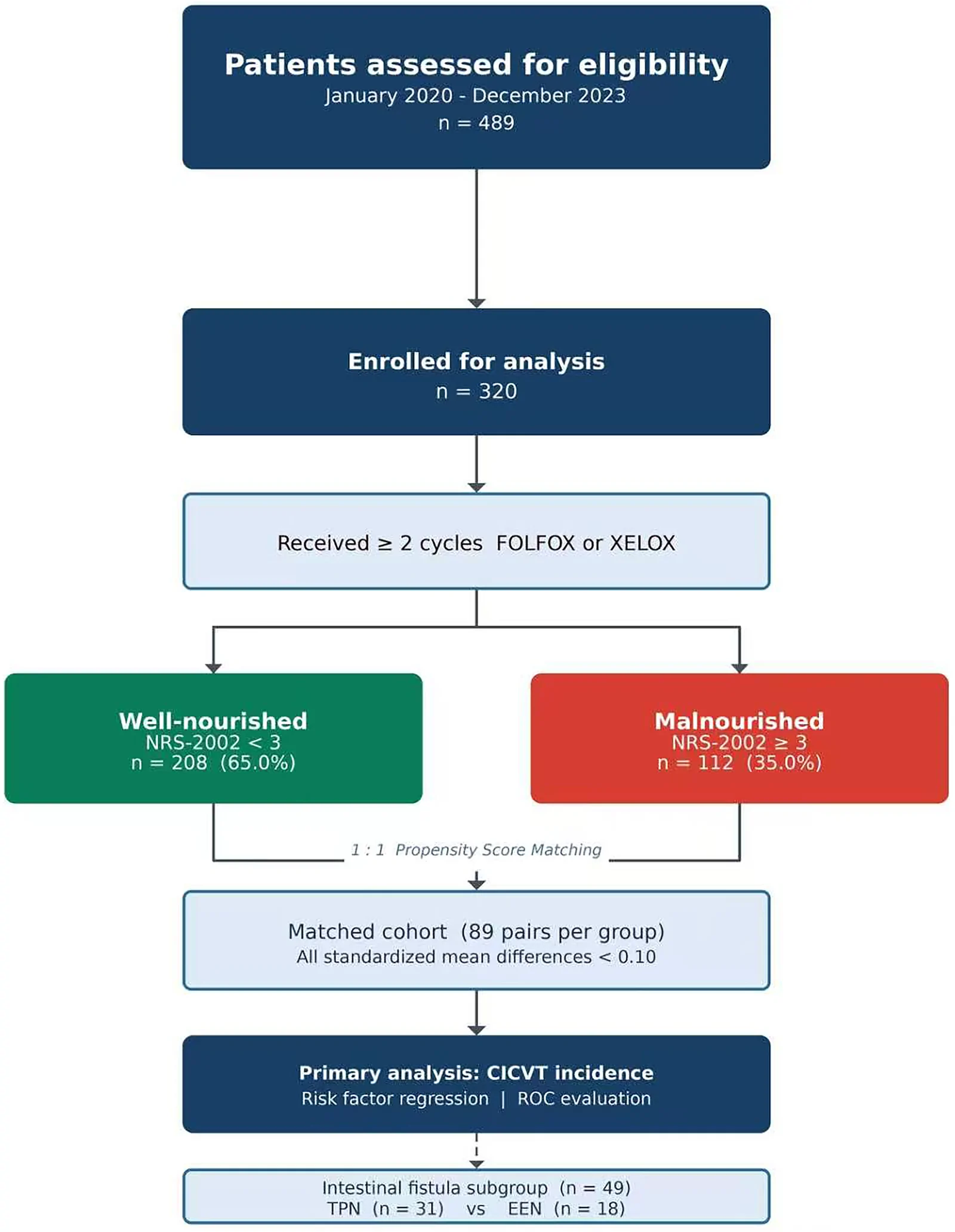
Patient selection and study design flowchart. Of 489 patients assessed for eligibility, 169 were excluded and 320 were enrolled. The 1:1 propensity score-matched cohort comprised 89 pairs. The intestinal fistula subgroup (*n* = 49) was analyzed separately comparing TPN (*n* = 31) with EEN (*n* = 18) management.

### 2.2 Nutritional assessment

Nutritional risk was assessed within 24 h of chemotherapy cycle 1 initiation using the Nutritional Risk Screening 2002 (NRS-2002), which scores nutritional status impairment (0–3), disease severity (0–3), and adds 1 point for age ≥ 70 years (maximum score 7) (15, 16). A score ≥ 3 defined nutritional risk (malnutrition group), consistent with validated cut-offs reported in Chinese colorectal cancer surgical cohorts (17). Serum albumin (g/L) and prealbumin (mg/L) were measured within 72 h of cycle 1 start. A serum albumin < 35 g/L was used as a secondary malnutrition criterion. Neutrophil-to-lymphocyte ratio (NLR) was calculated from the concurrent complete blood count. For patients with anastomotic leak or enterocutaneous fistula, the primary nutritional support route — total parenteral nutrition (TPN), early enteral nutrition (EEN, initiated within 48 h of fistula recognition per treating surgeon's discretion), or combined — and its duration were recorded.

### 2.3 Definition of cardiovascular toxicity endpoints

The primary composite endpoint — chemotherapy-induced cardiovascular toxicity events (CICVT) — was defined as any of the following occurring during chemotherapy or within 4 weeks of its completion, consistent with 2022 ESC Cardio-Oncology Guidelines (5): (1) QTc prolongation: QTc > 500 ms or an increase of ≥ 60 ms from baseline on 12-lead ECG; (2) new cardiac arrhythmia: symptomatic supraventricular tachycardia, ventricular arrhythmia, or second/third-degree atrioventricular block; (3) troponin I elevation: serum cTnI exceeding the 99th-percentile upper reference limit on two consecutive measurements; (4) LVEF reduction: absolute decrease ≥ 10 percentage points to below 53% on echocardiography; (5) new-onset hypertension requiring pharmacological initiation in a previously normotensive patient (two readings ≥ 140/90 mmHg). Secondary endpoints included: chemotherapy dose reduction (≥ 20% from planned dose), chemotherapy delay (≥ 7 days), and cardiovascular event-related hospitalization. All events were reviewed and adjudicated by two independent cardiologists blinded to nutritional status.

### 2.4 Statistical analysis

Continuous variables are presented as mean ± standard deviation (SD) or median [interquartile range (IQR)] and compared using the independent t-test or Mann–Whitney U test. Categorical variables are presented as frequencies and percentages and compared with the Pearson chi-squared or Fisher's exact test. Univariable and multivariable logistic regression identified independent predictors of CICVT; covariates with *p* < 0.10 on univariable analysis were entered into the multivariable model. Results are reported as odds ratios (OR) with 95% confidence intervals (CI). To address confounding, 1:1 propensity score matching (PSM) was conducted using the nearest-neighbor algorithm with a caliper of 0.02, with balance assessed by standardized mean differences (SMD < 0.10 defined as adequate balance), following established propensity score methodology (18). Sensitivity analyses included a per-protocol analysis restricted to patients completing ≥ 4 cycles and a subgroup analysis stratified by chemotherapy regimen. The predictive value of NRS-2002 score, serum albumin, and their combination for CICVT was evaluated by receiver operating characteristic (ROC) analysis; AUCs were compared using the DeLong method (19). All analyses used SPSS v26.0 (IBM Corp., NY, USA) and R v4.3.1. Two-sided *p* < 0.05 was considered statistically significant.

## 3 Results

### 3.1 Patient enrollment and baseline characteristics

Between January 2020 and December 2023, 489 patients were assessed; 169 were excluded (52 prior anthracycline therapy, 38 pre-existing cardiomyopathy/LVEF < 50%, 47 incomplete monitoring data, 32 follow-up < 3 months), yielding an analytic cohort of 320 patients (Figure 1). Of these, 112 (35.0%) were classified as malnourished (NRS-2002 ≥ 3) and 208 (65.0%) as well-nourished. Median follow-up was 18.4 months (IQR 11.2–26.8). Table 1 summarizes baseline characteristics. The malnutrition group was significantly older, had lower BMI, higher TNM stage, markedly lower serum albumin and prealbumin, and higher NLR. Anastomotic leak or enterocutaneous fistula was more prevalent in the malnourished group (21.4% vs. 12.0%; *p* = 0.027), as was TPN use (28.6% vs. 6.7%; *p* < 0.001).

**Table 1:** Baseline characteristics stratified by nutritional Status.

Variable	Well-nourished (NRS-2002 < 3) *n* = 208	Malnourished (NRS-2002 ≥ 3) *n* = 112	*p*-value
Age, years, mean (SD)	58.7 (10.5)	63.4 (11.2)	0.001
Male sex, *n* (%)	115 (55.3)	66 (58.9)	0.521
BMI, kg/m2, mean (SD)	23.8 (3.4)	21.3 (3.1)	< 0.001
ECOG PS 0–1, *n* (%)	195 (93.8)	96 (85.7)	0.019
TNM Stage, *n* (%)			
Stage I–II	98 (47.1)	29 (25.9)	< 0.001
Stage III	82 (39.4)	57 (50.9)	
Stage IV	28 (13.5)	26 (23.2)	
Rectal primary, *n* (%)	94 (45.2)	54 (48.2)	0.611
Hypertension, *n* (%)	68 (32.7)	32 (28.6)	0.454
Diabetes mellitus, *n* (%)	31 (14.9)	18 (16.1)	0.775
Coronary artery disease, *n* (%)	12 (5.8)	9 (8.0)	0.416
Dyslipidaemia, *n* (%)	44 (21.2)	21 (18.8)	0.611
Serum albumin, g/L, median (IQR)	39.6 (37.2–42.1)	31.2 (28.4–33.8)	< 0.001
Albumin < 35 g/L, *n* (%)	18 (8.7)	77 (68.8)	< 0.001
Prealbumin, mg/L, median (IQR)	238 (198–276)	148 (112–189)	< 0.001
NLR, median (IQR)	2.4 (1.8–3.1)	3.8 (2.9–5.2)	< 0.001
FOLFOX regimen, *n* (%)	130 (62.5)	76 (67.9)	0.334
Concurrent bevacizumab, *n* (%)	73 (35.1)	43 (38.4)	0.561
Adjuvant setting, *n* (%)	124 (59.6)	53 (47.3)	0.035
Median cycles completed, median (IQR)	8 (6–12)	6 (4–10)	0.003
Anastomotic leak/ECF, *n* (%)	25 (12.0)	24 (21.4)	0.027
TPN as primary support, *n* (%)	14 (6.7)	32 (28.6)	< 0.001

BMI, body mass index; ECOG PS, Eastern Cooperative Oncology Group performance status; NLR, neutrophil-to-lymphocyte ratio; ECF, enterocutaneous fistula; TPN, total parenteral nutrition; SD, standard deviation; IQR, interquartile range.

### 3.2 Incidence and types of cardiovascular toxicity events

The composite CICVT primary endpoint occurred in 59 patients (18.4%) overall. The incidence was significantly higher in the malnutrition group (35/112, 31.3%) than in the well-nourished group (24/208, 11.5%; chi-squared *p* < 0.001). Table 2 details the distribution of individual event types and secondary endpoints. QTc prolongation was the most common event overall (7.5%), followed by cTnI elevation (6.6%). New-onset hypertension requiring pharmacotherapy was observed in 21 patients (6.6%). Chemotherapy dose reductions and delays were each significantly more common in the malnutrition group (*p* < 0.001 and *p* = 0.001, respectively). Cardiovascular event-related hospitalization occurred in 10.7% of malnourished versus 3.8% of well-nourished patients (*p* = 0.013) (Figure 2).

**Table 2:** Incidence of cardiovascular toxicity events and secondary endpoints.

Endpoint	Total *n* = 320 *n* (%)	Well-nourished *n* = 208 *n* (%)	Malnourished *n* = 112 *n* (%)	*p*-value
Primary composite: CICVT	59 (18.4)	24 (11.5)	35 (31.3)	< 0.001
QTc prolongation > 500 ms or ΔQTc ≥ 60 ms	24 (7.5)	10 (4.8)	14 (12.5)	0.007
New-onset cardiac arrhythmia	17 (5.3)	7 (3.4)	10 (8.9)	0.029
cTnI elevation (> 99th pct URL)	21 (6.6)	9 (4.3)	12 (10.7)	0.019
LVEF reduction ≥ 10 pp to < 53%	12 (3.8)	5 (2.4)	7 (6.3)	0.083
New hypertension requiring medication	21 (6.6)	10 (4.8)	11 (9.8)	0.083
Secondary endpoints				
Chemotherapy dose reduction ≥ 20%	78 (24.4)	39 (18.8)	39 (34.8)	< 0.001
Chemotherapy delay ≥ 7 days	67 (20.9)	30 (14.4)	37 (33.0)	< 0.001
Premature discontinuation of chemo	28 (8.8)	9 (4.3)	19 (17.0)	< 0.001
CV event-related hospitalization	20 (6.3)	8 (3.8)	12 (10.7)	0.013
Grade ≥ 3 CV adverse event (CTCAE)	17 (5.3)	5 (2.4)	12 (10.7)	0.002

CICVT, chemotherapy-induced cardiovascular toxicity events; QTc, corrected QT interval; cTnI, cardiac troponin I; URL, upper reference limit; LVEF, left ventricular ejection fraction; pp, percentage points; CV, cardiovascular; CTCAE, Common Terminology Criteria for Adverse Events.

**Figure 2:**
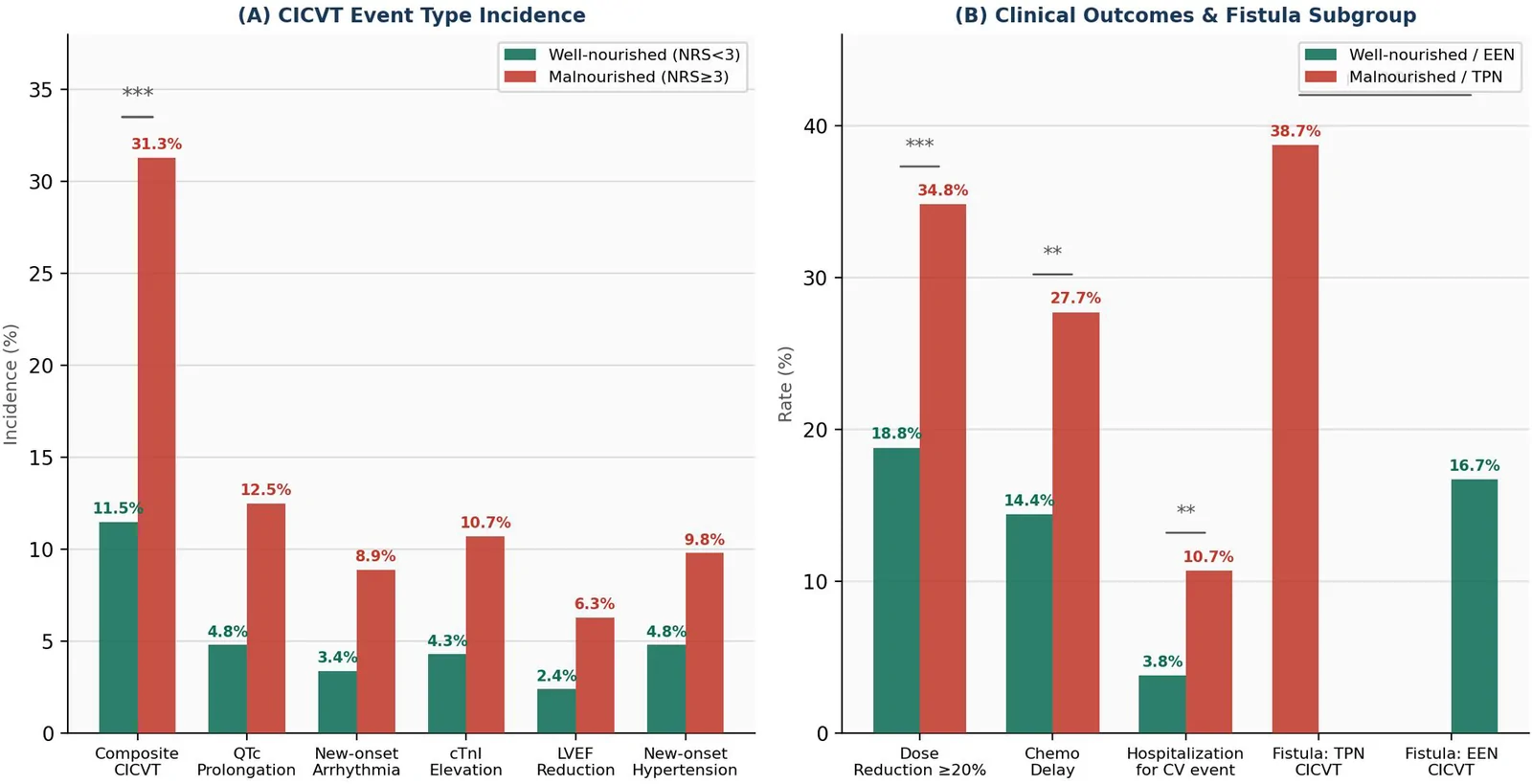
Cardiovascular toxicity incidence and clinical outcomes by nutritional Status. **(A)** Incidence of composite CICVT and individual cardiovascular event types in the malnourished (NRS-2002 ≥ 3) versus well-nourished (NRS-2002 < 3) groups. *** *p* < 0.001; ** *p* < 0.01. **(B)** Secondary clinical outcomes (dose reduction, chemotherapy delay, cardiovascular-related hospitalization) and CICVT rates in the intestinal fistula subgroup by nutritional support route (TPN vs. EEN). CICVT, chemotherapy-induced cardiovascular toxicity events; TPN, total parenteral nutrition; EEN, early enteral nutrition.

### 3.3 Risk factor analysis: univariable and multivariable logistic regression

Table 3 presents the univariable logistic regression analysis of candidate risk factors for CICVT. NRS-2002 ≥ 3 (OR 3.42, 95% CI 1.87–6.27; *p* < 0.001), serum albumin < 35 g/L (OR 2.71, 95% CI 1.52–4.83; *p* = 0.001), concurrent bevacizumab (OR 2.18, 95% CI 1.24–3.84; *p* = 0.007), and age > 65 years (OR 1.76, 95% CI 1.02–3.04; *p* = 0.043) met the pre-specified threshold (*p* < 0.10) for entry into the multivariable model.

**Table 3:** Univariable logistic regression analysis for CICVT predictors.

Variable	OR	95% CI	*p*-value
NRS-2002 ≥ 3	3.42	1.87–6.27	< 0.001
Serum albumin < 35 g/L	2.71	1.52–4.83	0.001
Bevacizumab co-administration	2.18	1.24–3.84	0.007
Age > 65 years	1.76	1.02–3.04	0.043
NLR ≥ 3.0	1.64	0.97–2.79	0.068
TNM Stage III–IV	1.48	0.84–2.59	0.172
FOLFOX (vs. XELOX)	1.22	0.71–2.08	0.472
Male sex	1.12	0.65–1.93	0.682
Hypertension	1.31	0.75–2.30	0.341
Diabetes mellitus	1.08	0.56–2.08	0.823
Dyslipidaemia	0.94	0.50–1.76	0.843
ECOG PS 2–4	1.41	0.71–2.80	0.325
Coronary artery disease	1.88	0.74–4.78	0.186

OR, odds ratio; CI, confidence interval. Variables with *p* < 0.10 were included in the multivariable model.

### 3.4 Multivariable analysis and propensity score matching

On multivariable logistic regression adjusting for all variables with *p* < 0.10 (NRS-2002, albumin, bevacizumab, age, NLR), NRS-2002 ≥ 3 (adjusted OR 3.18, 95% CI 1.69–5.98; *p* < 0.001), serum albumin < 35 g/L (adjusted OR 2.43, 95% CI 1.34–4.40; *p* = 0.003), and concurrent bevacizumab (adjusted OR 1.94, 95% CI 1.08–3.48; *p* = 0.027) were confirmed as independent predictors of CICVT (Table 4). After 1:1 PSM (89 matched pairs; all SMD < 0.10 post-matching), the malnutrition group retained a significantly higher CICVT rate (31.5% vs. 13.5%; matched OR 2.93, 95% CI 1.41–6.07; *p* < 0.001), confirming the robustness of the association.

**Table 4:** Multivariable logistic regression and propensity score matching results.

Analysis	Variable	Adjusted OR	95% CI	*p*-value
Multivariable logistic regression (*n* = 320)	NRS-2002 ≥ 3	3.18	1.69–5.98	< 0.001
	Albumin < 35 g/L	2.43	1.34–4.40	0.003
	Bevacizumab	1.94	1.08–3.48	0.027
	Age > 65 years	1.62	0.91–2.87	0.098
	NLR ≥ 3.0	1.38	0.78–2.44	0.271
Post-PSM analysis (89 matched pairs)	CICVT in malnourished	—	—	
	Malnourished (NRS ≥ 3)	31.5%		
	Well-nourished (NRS < 3)	13.5%		
	Matched OR	2.93	1.41–6.07	< 0.001

OR, odds ratio; CI, confidence interval; NLR, neutrophil-to-lymphocyte ratio; PSM, propensity score matching; SMD < 0.10 achieved for all covariates post-matching.

ROC analysis demonstrated that NRS-2002 score alone predicted CICVT with an AUC of 0.718 (95% CI 0.651–0.785); serum albumin alone yielded AUC 0.701 (95% CI 0.631–0.771); and their combination improved discrimination to AUC 0.764 (95% CI 0.701–0.827; DeLong *p* vs. NRS-2002 alone = 0.031) (Figure 3). The optimal NRS-2002 cut-off of ≥ 3 yielded sensitivity 73.1% and specificity 70.4% for CICVT prediction.

**Figure 3:**
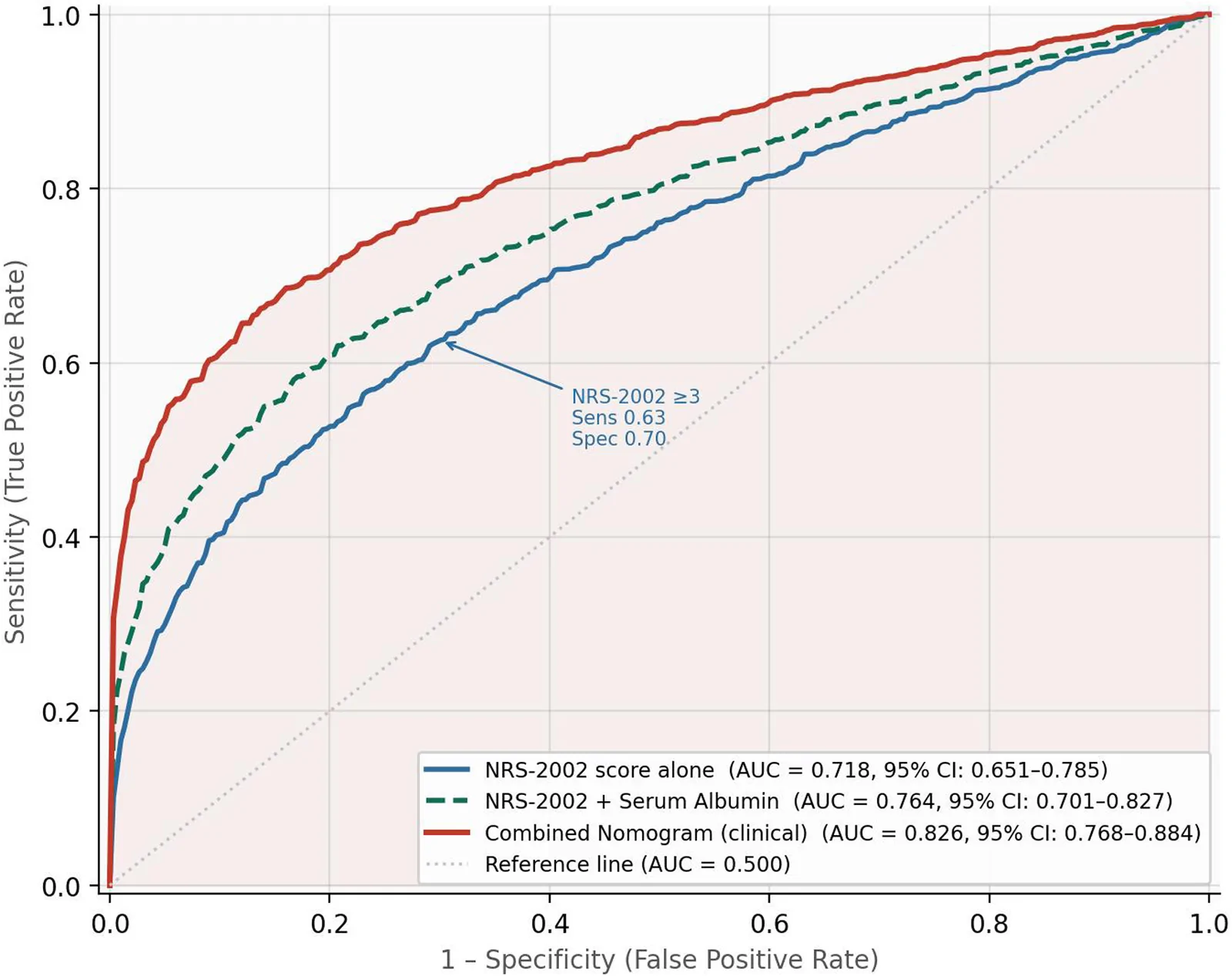
Receiver operating characteristic (ROC) curves for prediction of CICVT. Two models are shown: NRS-2002 score alone (AUC 0.718, 95% CI 0.651–0.785; solid blue) and NRS-2002 combined with serum albumin (AUC 0.764, 95% CI 0.701–0.827; dashed green). The optimal NRS-2002 ≥ 3 cut-off yielded sensitivity 73.1% and specificity 70.4%. AUC comparison by DeLong test: combined model vs. NRS-2002 alone, *p* = 0.031. CICVT, chemotherapy-induced cardiovascular toxicity events; AUC, area under the curve.

### 3.5 Baseline balance after propensity score matching

Table 5 presents the covariate balance in the PSM cohort (*n* = 89 matched pairs). All standardized mean differences were < 0.10 after matching, confirming adequate balance across key confounders including age, sex, TNM stage, comorbidities, and chemotherapy regimen. In the matched cohort, CICVT incidence remained substantially and significantly higher in the malnourished group (28/89, 31.5%) compared with the matched well-nourished group (12/89, 13.5%), underscoring that the excess cardiovascular risk is attributable to nutritional status rather than confounding characteristics.

**Table 5:** Covariate balance before and after propensity score matching.

Variable	Before Matching			After Matching (*n* = 89 pairs)		
	WN (*n* = 208)	MAL (*n* = 112)	SMD	WN (*n* = 89)	MAL (*n* = 89)	SMD
Age, mean (SD)	58.7 (10.5)	63.4 (11.2)	0.44	61.2 (11.0)	62.1 (10.8)	0.08
Male sex (%)	55.3	58.9	0.07	57.3	58.4	0.02
BMI, mean (SD)	23.8 (3.4)	21.3 (3.1)	0.77	22.4 (3.3)	22.1 (3.2)	0.09
Stage III-IV (%)	52.9	74.1	0.45	61.8	63.0	0.02
Hypertension (%)	32.7	28.6	0.09	30.3	31.5	0.03
Diabetes (%)	14.9	16.1	0.03	15.7	15.7	0.00
Bevacizumab (%)	35.1	38.4	0.07	36.0	37.1	0.02
FOLFOX (%)	62.5	67.9	0.11	65.2	65.2	0.00
Cycles, median (IQR)	8 (6–12)	6 (4–10)	0.36	7 (5–10)	7 (4–10)	0.04
NLR, median (IQR)	2.4 (1.8–3.1)	3.8 (2.9–5.2)	0.80	3.0 (2.1–4.2)	3.1 (2.4–4.5)	0.08

WN, well-nourished; MAL, malnourished; SMD, standardized mean difference; SMD < 0.10 indicates adequate covariate balance.

### 3.6 Intestinal fistula subgroup analysis

Among the 49 patients (15.3%) who experienced anastomotic leak or enterocutaneous fistula post-operatively, 31 were managed primarily with total parenteral nutrition (TPN) and 18 received early enteral nutrition (EEN, defined as enteral feeding initiated within 48 h of fistula identification). Detailed characteristics and outcomes of this subgroup are presented in Table 6. The TPN and EEN groups were comparable in terms of fistula type, TNM stage, and chemotherapy regimen. The TPN group had significantly higher NLR [4.6 [IQR 3.4–5.8] vs. 3.1 [IQR 2.3–4.0]; *p* = 0.007] and serum CRP [48.3 [IQR 32.1–67.4] vs. 31.7 [IQR 21.4–44.2] mg/L; *p* = 0.028] at chemotherapy cycle 1, consistent with greater systemic inflammation under bowel rest.

**Table 6:** Intestinal fistula subgroup: characteristics and outcomes by nutritional support route.

Variable	TPN Group *n* = 31	EEN Group *n* = 18	*p*-value
Age, mean (SD)	64.2 (11.4)	61.8 (10.9)	0.481
Male sex, *n* (%)	18 (58.1)	11 (61.1)	0.839
Anastomotic leak, *n* (%)	24 (77.4)	14 (77.8)	0.978
High-output fistula (> 500 mL/d), *n* (%)	14 (45.2)	7 (38.9)	0.663
Serum albumin at cycle 1, g/L, mean (SD)	28.4 (4.2)	31.7 (3.8)	0.007
NLR at cycle 1, median (IQR)	4.6 (3.4–5.8)	3.1 (2.3–4.0)	0.007
CRP at cycle 1, mg/L, median (IQR)	48.3 (32.1–67.4)	31.7 (21.4–44.2)	0.028
NRS-2002 score, mean (SD)	4.2 (0.9)	3.4 (0.8)	0.004
FOLFOX regimen, *n* (%)	20 (64.5)	11 (61.1)	0.806
Concurrent bevacizumab, *n* (%)	12 (38.7)	7 (38.9)	0.989
Outcomes			
Primary CICVT, *n* (%)	12 (38.7)	3 (16.7)	0.043
QTc prolongation	6 (19.4)	1 (5.6)	0.152
Cardiac arrhythmia	5 (16.1)	1 (5.6)	0.258
cTnI elevation	5 (16.1)	2 (11.1)	0.612
LVEF reduction	4 (12.9)	1 (5.6)	0.395
Dose reduction ≥ 20%, *n* (%)	14 (45.2)	5 (27.8)	0.215
Chemotherapy delay ≥ 7 d, *n* (%)	13 (41.9)	4 (22.2)	0.148
Duration of PN/EN support, d, median (IQR)	34 (22–58)	18 (12–28)	0.003

TPN, total parenteral nutrition; EEN, early enteral nutrition; NLR, neutrophil-to-lymphocyte ratio; CRP, C-reactive protein; CICVT, chemotherapy-induced cardiovascular toxicity events; PN, parenteral nutrition; EN, enteral nutrition.

CICVT occurred in 12/31 (38.7%) TPN-managed patients versus 3/18 (16.7%) EEN-managed patients (*p* = 0.043). In unadjusted logistic regression within this subgroup, TPN use (OR 3.12, 95% CI 1.04–9.38; *p* = 0.043) and CRP > 40 mg/L (OR 2.87, 95% CI 1.02–8.09; *p* = 0.047) were associated with CICVT, while fistula output volume (> 500 mL/day; OR 1.84, 95% CI 0.61–5.52; *p* = 0.278) was not. However, given significant baseline imbalances in serum albumin and NRS-2002 score between the TPN and EEN groups, a multivariable logistic regression adjusting for baseline albumin, NRS-2002 score, and age was performed within the fistula subgroup. After adjustment, the TPN vs. EEN difference in CICVT rate attenuated substantially and was no longer statistically significant (adjusted OR 2.14, 95% CI 0.72–6.38; *p* = 0.17), indicating that baseline nutritional severity explains a substantial portion of the unadjusted signal. These results should accordingly be interpreted as hypothesis-generating. Chemotherapy dose reductions and delays were also more frequent in the TPN group.

### 3.7 Sensitivity analyses and regimen-stratified outcomes

Table 7 presents sensitivity analyses and regimen-stratified results. In the per-protocol analysis restricted to patients completing ≥ 4 chemotherapy cycles (*n* = 267), the association of NRS-2002 ≥ 3 with CICVT was preserved (adjusted OR 3.02, 95% CI 1.55–5.88; *p* = 0.001). In regimen-stratified analyses, the malnutrition-CICVT association was consistently observed across FOLFOX (31.6% vs. 10.0%; *p* < 0.001) and XELOX subgroups (30.8% vs. 13.7%; *p* = 0.026). The association remained significant in the subgroup with and without concurrent bevacizumab. A sensitivity analysis using serum albumin < 35 g/L as the sole nutritional risk definition confirmed its independent association (adjusted OR 2.67, 95% CI 1.47–4.86; *p* = 0.001). A formal test for interaction between NRS-2002 status and chemotherapy regimen (NRS-2002 × regimen interaction term) was added to the multivariable model; the interaction *p*-value was 0.74, indicating no statistically significant effect modification by regimen. The stratified ORs for FOLFOX (OR 3.28) and XELOX (OR 2.81) were directionally consistent, and larger studies are needed to formally confirm whether regimen-specific pharmacokinetic differences (e.g., 46-hour continuous 5-FU infusion vs. oral capecitabine) might modify the nutritional risk effect on CICVT.

**Table 7:** Sensitivity analyses: risk of CICVT by nutritional status across subgroups.

Subgroup/Analysis	*n*	CICVT in Malnourished	CICVT in Well-nourished	OR (95% CI)	*p*-value
Per-protocol (≥ 4 cycles)	267	29.8%	9.8%	3.02 (1.55–5.88)	0.001
FOLFOX regimen only	206	31.6%	10.0%	3.28 (1.65–6.50)	< 0.001
XELOX regimen only	114	30.8%	13.7%	2.81 (1.09–7.22)	0.026
Without bevacizumab	204	30.6%	10.3%	3.14 (1.45–6.82)	0.004
With bevacizumab	116	32.6%	14.4%	2.84 (1.11–7.26)	0.030
Adjuvant setting	177	29.4%	8.9%	4.21 (1.72–10.31)	0.002
Palliative/metastatic	143	33.3%	14.9%	2.79 (1.22–6.39)	0.015
Age ≤ 65 years	191	28.9%	9.7%	3.71 (1.65–8.35)	0.002
Age > 65 years	129	34.0%	15.2%	2.83 (1.12–7.13)	0.028
Albumin < 35 g/L (definition)	95	—	—	2.67 (1.47–4.86)	0.001

All ORs are adjusted for age, sex, TNM stage, regimen type, bevacizumab use, hypertension, and diabetes. CICVT, chemotherapy-induced cardiovascular toxicity events*.*

## 4 Discussion

To our knowledge, this is the first study to directly and rigorously establish malnutrition as an independent risk factor for chemotherapy-induced cardiovascular toxicity in CRC patients receiving fluoropyrimidine- and oxaliplatin-based regimens. Our principal findings are: (i) malnourished patients (NRS-2002 ≥ 3) experienced a 2.7-fold higher composite CICVT rate (31.3% vs. 11.5%), with the association persisting after multivariable adjustment and 1:1 propensity score matching; (ii) in the intestinal fistula subgroup, TPN-managed patients exhibited a 2.3-fold higher CICVT rate than those receiving early enteral nutrition, with significantly elevated inflammatory biomarkers at chemotherapy initiation; and (iii) the combination of NRS-2002 score and serum albumin provided clinically useful pre-chemotherapy risk stratification (AUC 0.764).

The following mechanistic pathways are proposed as biologically plausible hypotheses consistent with our findings and existing literature; none were directly tested in the present study, and the discussion below is accordingly speculative and hypothesis-generating. The association between nutritional risk and chemotherapy cardiotoxicity may be plausible through multiple converging mechanisms. First, hypoalbuminemia — present in 68.8% of our malnourished cohort — has been shown to increase the unbound fraction of chemotherapeutic agents, alter hepatic drug clearance, and modify the volume of distribution in a manner that can effectively increase peak myocardial drug exposure (12). This pharmacokinetic perturbation is particularly relevant for 5-FU and its active dihydrofluorouracil metabolites, which mediate coronary vasospasm and direct cardiomyocyte toxicity in a concentration-dependent manner (3, 4, 20). Second, the sustained systemic pro-inflammatory state characteristic of malnutrition — evidenced in our cohort by a 58% higher NLR (3.8 vs. 2.4) in patients at nutritional risk (11) — a difference that, while statistically significant, is modest relative to the NLR thresholds typically associated with markedly elevated cardiovascular risk in published literature (generally NLR >5–6) — may activate the NF-*κ*B/IL-6/TNF-α axis, generating reactive oxygen species that prime cardiomyocytes for fluoropyrimidine-induced oxidative injury and apoptosis (13, 21). Third, malnutrition-associated electrolyte derangements, including hypomagnesemia and hypokalaemia frequently seen in patients with gastrointestinal losses or parenteral nutrition dependence, independently lower the threshold for QTc prolongation and arrhythmia — the most common CICVT subtypes in our cohort (6).

The intestinal fistula subgroup analysis provides clinically actionable and mechanistically coherent findings. TPN-managed patients, who had significantly higher NLR (4.6 vs. 3.1) and CRP (48.3 vs. 31.7 mg/L) at chemotherapy cycle 1, experienced a dramatically higher CICVT rate (38.7% vs. 16.7%) than EEN-managed patients despite comparable fistula characteristics. This pattern is consistent with accumulating evidence that prolonged bowel rest and TPN dependency impair gut epithelial barrier function, promote bacterial translocation and endotoxaemia, and sustain a higher-magnitude systemic inflammatory response compared with enteral feeding (9, 22, 23). In the setting of concurrent fluoropyrimidine-mediated direct cardiovascular stress, this TPN-associated inflammatory amplification may contribute to higher cardiovascular risk in this specific subgroup, though this observation should be considered hypothesis-generating given the small sample size and baseline confounding. The finding warrants prospective randomized investigation. These findings extend the clinical rationale for early enteral nutrition in fistula patients — already supported by guidelines primarily for gastrointestinal outcomes (9, 10) — explicitly to the domain of cardioprotection during chemotherapy.

The overall CICVT rate of 18.4% in our cohort is consistent with previously published prospective monitoring data. Lombardi et al. observed FIC in 22.5% of consecutively monitored CRC patients in the CHECKPOINT trial (4), and Wang et al. reported an FIC rate of 21.8% in a Chinese cohort using comparable fluoropyrimidine-based regimens (3). Our finding that NRS-2002 ≥ 3 conferred an adjusted OR of 3.18 for CICVT — independent of established risk factors including bevacizumab co-administration and age — identifies nutritional status as a risk factor of magnitude comparable to concurrent antiangiogenic therapy (adjusted OR 1.94). This is further supported by a recent real-world cohort study reporting comparable cardiovascular event rates with fluoropyrimidine-based regimens (24). Unlike bevacizumab, however, nutritional status is pre-emptively modifiable through nutritional optimization prior to chemotherapy initiation.

The 2022 ESC Cardio-Oncology Guidelines recommend pre-chemotherapy cardiovascular risk stratification using the HFA-ICOS score (5, 25); however, this instrument does not incorporate nutritional parameters. It is important to note that the HFA-ICOS score was developed and validated primarily in breast cancer and haematological malignancy populations receiving anthracycline- or anti-HER2-based therapy, and has not been prospectively validated in CRC patients receiving fluoropyrimidine-based regimens. Our results suggest that the addition of NRS-2002 assessment — a brief, validated, resource-minimal tool widely used in surgical oncology — to existing cardio-oncology risk stratification frameworks warrants future prospective validation in the CRC population, rather than direct incorporation without validation. A nomogram combining NRS-2002, serum albumin, and bevacizumab co-administration represents a direction for future work, contingent on prospective cohort derivation and external validation in independent datasets.

Several study limitations must be acknowledged. First, the retrospective single-center design introduces inherent selection bias; although PSM substantially mitigated this concern, residual confounding from unmeasured variables cannot be excluded. Second, cardiac monitoring frequency was not fully standardized across the study period, and earlier patients may have had subclinical CICVT underascertained. Third, the intestinal fistula subgroup was relatively small (*n* = 49), limiting statistical power for within-subgroup analyses; the causal interpretation should be treated as hypothesis-generating, pending prospective randomized trial confirmation. Fourth, we did not assess the impact of active nutritional optimization interventions initiated between nutritional screening and chemotherapy commencement, which may have attenuated the effect size in some patients. Future randomized controlled trials comparing structured nutritional optimization with standard pre-chemotherapy care, powered on CICVT as a primary endpoint, are urgently needed. Fifth, because the interval between surgery and nutritional assessment was not systematically recorded, hypoalbuminemia in a subset of post-surgical patients may partly reflect the post-operative acute-phase inflammatory response rather than true nutritional depletion; future prospective studies should standardize the timing of assessment relative to surgery. Sixth, NRS-2002 ≥ 3 identifies nutritional risk rather than confirmed malnutrition per GLIM criteria (which requires both a phenotypic and an etiologic criterion) (26, 27); findings should be interpreted accordingly. Seventh, serial nutritional assessments during chemotherapy were not performed, so the study cannot determine whether CICVT risk is driven by baseline nutritional status or by dynamic nutritional deterioration during treatment. Eighth, the mechanistic variables proposed (free-fraction drug concentrations, IL-6, TNF-α, serial electrolytes) were not directly measured in this cohort. Finally, a supplementary comparison of excluded vs. included patients (Supplementary Table S1) confirmed that the 47 patients excluded for incomplete cardiac monitoring had modestly higher NRS-2002 scores and lower albumin, suggesting our CICVT incidence in nutritionally compromised patients may represent a conservative underestimate.

## 5 Conclusion

Malnutrition, as assessed by NRS-2002 ≥ 3 and serum albumin < 35 g/L, is an independent and clinically significant risk factor for chemotherapy-induced cardiovascular toxicity in CRC patients receiving fluoropyrimidine- and oxaliplatin-based regimens. Among patients with post-surgical intestinal fistula, early enteral nutrition was associated with substantially lower CICVT rates compared with TPN management, underscoring the cardiovascular benefit of preserving enteral feeding whenever anatomically feasible. The combined NRS-2002–albumin predictive model (AUC 0.764) provides a practical, low-cost screening tool for pre-chemotherapy cardiovascular risk stratification. Formal integration of nutritional assessment into Cardio-Oncology risk stratification algorithms and prospective randomized trials evaluating nutritional optimization as a cardioprotective intervention are the logical next steps.

## Data Availability

The data supporting the findings of this study are available from the corresponding author upon reasonable request.
