# Supplementary material for: Malnutrition as an independent risk factor for fluoropyrimidine- and oxaliplatin-based chemotherapy-induced cardiovascular toxicity in colorectal cancer: a single-center retrospective cohort study
Source: Front Cardiovasc Med. 2026 Sep 21;13:1936804. doi: 10.3389/fcvm.2026.1936804 (PMC13634806; doi:10.3389/fcvm.2026.1936804)
Supplement: Supplementary file 1 [file Table1.docx]

**Supplementary Table S1. Comparison of included patients and patients excluded because of incomplete cardiac monitoring**

**Source note:** The uploaded author-proof PDF cites Table S1 but does not contain the numerical supplementary data for the 47 excluded patients. The placeholders below must be replaced with values from the original dataset before journal submission.

| **Variable** | **Included cohort (n = 320)** | **Excluded: incomplete cardiac monitoring (n = 47)** | **p-value** |
| --- | --- | --- | --- |
| Patients, n | 320 | 47 | - |
| NRS-2002 score, median (IQR) | Not reported in proof | Not reported in proof (manuscript states: modestly higher) | Not reported |
| NRS-2002 ≥ 3, n (%) | 112 (35.0) | Not reported in proof | Not reported |
| Serum albumin, g/L, median (IQR) | Not reported in proof | Not reported in proof (manuscript states: lower) | Not reported |
| Serum albumin < 35 g/L, n (%) | 95 (29.7) | Not reported in proof | Not reported |

**Abbreviations:** NRS-2002, Nutritional Risk Screening 2002; IQR, interquartile range.

**Proof-supported interpretation:** the 47 patients excluded for incomplete cardiac monitoring had modestly higher NRS-2002 scores and lower serum albumin than the included cohort. Exact statistics are not present in the proof PDF.
